# The Impact of Exposure Profile on the Efficacy of Dual Amylin and Calcitonin Receptor Agonist Therapy

**DOI:** 10.3390/biomedicines10102365

**Published:** 2022-09-22

**Authors:** Nina Sonne, Anna Thorsø Larsen, Morten Asser Karsdal, Kim Henriksen

**Affiliations:** 1 Nordic BioscienceA/S, Department of Endocrinology, Herlev Hovedgade 205-207, 2730 Herlev, Denmark; 2 KeyBioscience AG, 6370 Stans, Switzerland

**Keywords:** DACRA, GPCR, obesity, type 2 diabetes, preclinical

## Abstract

Background: Dual Amylin and Calcitonin Receptor Agonists (DACRAs) are treatment candidates for obesity and type 2 diabetes. Recently, a once-weekly DACRA (KBP-A) showed promise, potentially due to its different exposure profile compared to daily DACRA (KBP). Parathyroid hormone, a G-protein-coupled receptor (GPCR) class B agonist, is an example of the exposure profile being critical to the effect. Since KBP and KBP-A also activate GPCR class B, we compared the effects of injection to continuous infusion of short-acting KBP and long-acting KBP-A in obese and diabetic rats to shed light on the role of exposure profiles. Methods: To explore the metabolic benefits of dose optimization, the following dosing profiles were compared in High Fat Diet (HFD)-fed Sprague–Dawley rats and diabetic Zucker Diabetic Fatty (ZDF) rats: (1) KBP dosed once-daily by injection or by continuous infusion in HFD and ZDF rats; (2) KBP injected once-daily and KBP-A injected once every 3rd day (Q3D) in HFD rats; (3) KBP-A injected Q3D or by infusion in ZDF rats. Results: KBP and KBP-A, delivered by either injection or infusion, resulted in similar weight and food intake reductions in HFD rats. In ZDF rats, injection of KBP improved glucose control significantly compared to infusion, while delivery of KBP-A by injection and continuous infusion was comparable in terms of glucose control. Conclusion: different dosing profiles of KBP and KBP-A had no impact on metabolic benefits in HFD rats. In diabetic ZDF rats, KBP by injection instead of infusion was superior, while for KBP-A the effects were similar.

## 1. Background

Amylin is a small peptide produced in the beta-cells of the pancreas and co-secreted with insulin [1]. In a physiological setting, amylin regulates food intake, body weight, and adiposity [2,3,4,5] and aids in controlling postprandial blood glucose by slowing the gastric emptying rate [5]. Pharmacological investigations reveal that these effects can be amplified, resulting in temporary food suppression, sustained weight loss, as well as improved glucose tolerance [6,7]. However, amylin lacks potency in terms of weight loss and glucose tolerance, and only one amylin analogue, pramlintide, has been FDA-approved and only as an add-on to insulin in people with diabetes (type 1 and 2). 

Dual amylin and calcitonin receptor agonists (DACRAs) are a novel group of peptides that potently activate both the calcitonin receptor, a G-protein-coupled receptor (GPCR), and the amylin receptor (consisting of the calcitonin receptor in association with Receptor Activity Modifying Protein (RAMP) 1, 2 or 3) [8,9]. In obese rats, DACRA treatment results in significant weight loss and improved post-prandial glucose control, which is partially mediated by a delay in gastric emptying [7,10]. In diabetic rats, DACRAs also improve fasting blood glucose and HbA1c [11,12]. In both models, the DACRA effects are superior to amylin treatment [7,13]. 

The first designed DACRAs, termed KBPs, were originally developed for once-daily administration and displayed an acute dose-dependent and prolonged action of 24–48 h food suppression, despite a pharmacokinetic (PK) profile peaking 10–20 min post s.c. injection and with total clearance after 2 h [9,14,15]. Recently, long-acting KBPs (termed KBP-As) developed for once-weekly administration in humans have been published [16]. The extended half-life of the peptides results from their conjugation to an acylation that binds to serum albumin, thereby allowing a slow release and clearance. Hence, KBP-As display a different PK and pharmacodynamic (PD) profile compared to non-acylated KBPs [16]. Whether the PD effects of KBP and KBP-A can be modulated by alternative exposure profiles is largely unexplored. 

Importantly, studies of another GPCR class B ligand, parathyroid hormone (PTH), and its receptor have shown that exposure duration is critical to the effect [17,18,19]. Intermittent exposure to PTH results in increased bone formation, whereas continuous infusion of PTH has the opposite effect [20,21,22]. This underlines the importance of a thorough understanding of the relationship between exposure and effect when dosing with KBP and KBP-A, which inherently has different plasma half-lives and exposure profiles. 

**Figure 1:**
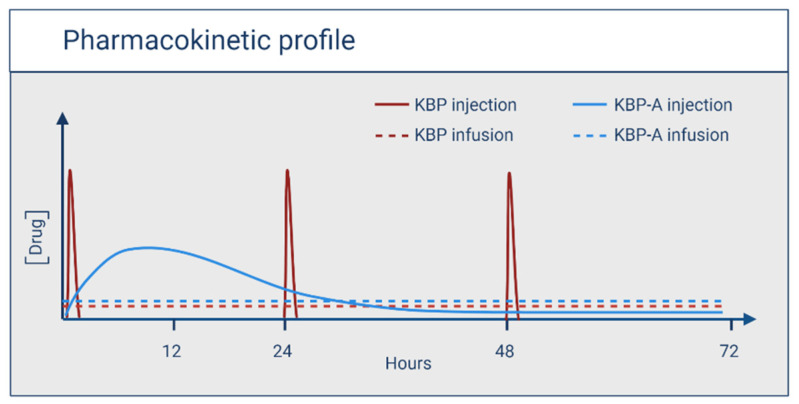
Schematic illustration of the pharmacokinetic profiles of KBP and KBP-A dosed by injection or infusion. Created with Biorender.com.

Based on the experience from PTH, we sought to determine the relationship between different dosing profiles and PD for KBP and KBP-A. Therefore, the consequence of different dosing profiles, including injection and infusion of KBP and KBP-A, was investigated as illustrated in (Figure 1). 

## 2. Methods

### 2.1. Peptide Therapy

The following DACRAs were used in the studies: KBP-042, KBP-088, and its acylated counterpart KBP-088A, and the acylated KBP-066A (all peptides: SynPeptide, Shanghai, China). Sequences of all peptides are shown in Table 1. All peptides activate the amylin and calcitonin receptor [9,11,16] with similar potency. Small potency differences can be compensated in vivo by increasing the dose. Pharmacologically, the difference in the peptides resides in the absence (KBP) or presence (KBP-A) of an acylation. The full names of the peptides are stated in the Materials and Methods section for transparency, while abbreviated names (KBP and KBP-A) are used in the Results section for simplicity. KBP-042 belongs to the first generation of KBPs. Later, KBP-088 and KBP-066 were designed and since their acylated counterparts. KBP-066A exhibits optimized stability at 37 °C making it suitable for continuous dosing via Alzet osmotic pumps (AgnTho’s, Lidingö, Sweden), as the drug-containing pump is placed subcutaneously at body temperature. Stability tests of KBP-042, −088, and −066A at 37 °C are described in the Supplementary section and shown in Supplementary Figures S2–S5. KBP-042 was dissolved in mannitol (5%, pH 4), while all other peptides were dissolved in saline (0.9%) for s.c. injection or continuous infusion. The dose ranges chosen for KBP-042, KBP-088, −088A, and −066A, were based on previous studies [7,9,14,16,23]. A higher drug dose was used in ZDF rat studies when using Alzet osmotic pumps, as these studies were carried out while the rats were still young and in a progressive growth phase. The pumps release a fixed amount of drug, and it is hence not possible to adjust the drug dose when the body weight increases.

### 2.2. Animal Experiments 

All animal procedures were performed in agreement with guidelines from the Animal Welfare Division of the Danish Ministry of Justice under the institutional license issued to Nordic Bioscience (2016-15-0201-00910) and were carried out in accordance with the ARRIVE guidelines. Animals were housed pairwise in enriched standard type IV cages (Scanbur A/S, Karlslunde, Denmark) under controlled temperature (21–23 °C, 55–65% relative humidity) and a 12-h light–dark cycle (lights on at 7:00 a.m.) with ad libitum access to food and water. All animal studies are described below, and the study designs are depicted in the schematic diagrams in Supplementary Figure S1.

### 2.3. Study (1) Infusion vs. Injection of KBP-042 in HFD-Fed Sprague–Dawley Rats

Male Sprague–Dawley rats (Envigo, Venray, The Netherlands) were obtained at 5–6 weeks of age and were fed a 60 kcal% fat high-fat diet (HFD) (#D12492, ResearchDiets, New Brunswick, NJ, USA) from arrival and throughout the study period. After 11 weeks on HFD, the rats were allocated to treatment groups according to body weight (average: 476 g ± 18 SD, n = 8 rats/ treatment group). The three treatment groups were: vehicle (saline) and KBP-042 (1.5 nmol/kg). These were delivered by two different approaches: by s.c. injection (INJ.) once daily, or by continuous infusion (INF.) using Alzet osmotic pumps (model 2004; AgnTho’s, Lidingö, Sweden). The osmotic infusion pump delivers a small amount of drug continuously, thereby assuring a constant exposure accumulating to 1.5 nmol/kg/day. All groups received an s.c. injection once daily of either saline solution (vehicle and KBP-042 INF.) or drug (KBP-042 INJ.) to ensure equal handling. On the day of the study, all rats underwent surgery (KBP-042 INF.) or SHAM surgery (vehicle and KBP-042 INJ.). The infusion pump was placed subcutaneously in the neck region. Isoflurane was used as anaesthesia, marcaine as local analgesia, and carprofen as systemic analgesia and anti-inflammatory treatment for 3 days. The treatment period lasted for 26 days. Bodyweight and food intake were monitored daily for the first 5 days, then every 3rd day. At study end, an oral glucose tolerance test (OGTT) (2 g/kg, 16 h fasted) was performed.

### 2.4. Study (2) Infusion vs. Injection of KBP-088 in Diabetic ZDF Rats

Male Zucker diabetic fatty (*fa/fa)* (ZDF) rats (Charles River Laboratories, Lyon, France) were obtained at 5–6 weeks of age and were fed on the Purina Laboratory Diet (# 5008, Brogaarden, Denmark) from arrival and throughout the study. The rats developed diabetes before they were allocated to treatment groups primarily based on fasting (6 h) blood glucose (FBG) (average: 15 mM ± 6 SD) and secondarily on body weight (average: 341 g ± 18 SD). The three treatment groups were: vehicle (saline, *n* = 8), and KBP-088 (2 nmol/kg), delivered by two different approaches: by s.c. injection (INJ., *n* = 7) once daily or by continuous infusion (INF., *n* = 8) using Alzet osmotic pumps (model 2004; AgnTho’s, Lidingö). All groups received an s.c. injection once daily of either saline (vehicle and KBP-088 INF.) or drug (KBP-088 INJ.) to ensure equal handling. On the first day of the study (10–11 weeks of age), all rats underwent surgery (KBP-088 INF) or SHAM surgery (vehicle and KBP-088 INJ.) as described in study (1). 

The pump was replaced with a new one on day 29 as described in (1) and vehicle and INJ. underwent SHAM. As the infusion pumps release a fixed amount of drug, all dosing was based on the weight on days −2, and 28 to ensure that INF. vs. INJ. received an equal amount of moles. Food intake and body weight were recorded once daily for one week following surgery/SHAM, then once weekly. Fasting blood glucose (6 h) was measured weekly, and HbA1c at the study end. After 7.5 weeks of treatment, an OGTT (1 g/kg) was performed in 11 h fasted rats. One vehicle rat died following the OGTT and the last FBG measurement (7 weeks treatment) was carried forward to study end (8 weeks treatment). 

### 2.5. Study (3) Comparison of KBP-088 and KBP-088A in HFD-Fed Sprague–Dawley Rats 

Male Sprague–Dawley rats (Envigo, Venray, The Netherlands) were obtained at 5–6 weeks of age and were fed a 60 kcal% fat high-fat diet (HFD) (#D12492, ResearchDiets, New Brunswick, NJ, USA) from arrival and throughout the study period. After 12 weeks on HFD, the rats were allocated to treatment groups according to body weight (average: 466 g ± 35 SD, *n* = 8 rats/ treatment group). The rats were treated for 9 weeks by s.c. injections of vehicle (saline), once-daily KBP-088 (1.5 nmol/kg QD) or KBP-088A every third day (4.5 nmol/kg Q3D). Data from the vehicle group and KBP-088 QD has been published previously [23] but is included here as a reference for KBP-088A Q3D. Bodyweight and food intake were monitored daily throughout the study, but KBP-088 QD and vehicle are only shown as weekly from day 14 for simplicity. After 8 weeks of treatment, an OGTT (2 g/kg, 16 h fasted) was performed 24 h after drug injection. At the study’s end, the rats were euthanized by exsanguination followed by dissection. Epididymal, perirenal, and subcutaneous inguinal fat depots were surgically removed and weighed. 

### 2.6. Study (4) Intravenous Glucose Tolerance Test in HFD-Fed Sprague–Dawley Rats

The study was similar to the one described in study (3). Male Sprague–Dawley (Taconic, Ejby, Denmark) rats arrived at 5–6 weeks of age and were fed a 60 kcal% fat high-fat diet (HFD) (#D12492, ResearchDiets, New Brunswick, NJ). At 15–16 weeks of age, the rats were allocated to treatment groups based on body weight (average: 554 g ± 55 SD, *n* = 10 rats/treatment group). The same treatment groups and frequency of weight and food monitoring were applied for this study as described in (3). The study duration was 37 days. Two intravenous glucose tolerance tests (IV-GTT) were performed on days 21 and 37. Prior to the IV-GTTs, both KBP groups were pre-dosed with 4.5 nmol/kg regardless of the “normal” dose concentration. This was to rule out the influence of dose differences (KBP-088 1.5 nmol/kg QD vs. KBP-088A 4.5 nmol/kg Q3D). Pre-dosing with KBP and KBP-A were performed −35 min and 24 h prior to the glucose challenge to investigate the relationship between glucose tolerance and a rapid (KBP-088) versus slow (KBP-088A) plasma peak. 

### 2.7. Study (5) Infusion vs. Injection of KBP-066A in Diabetic ZDF Rats

KBP-066A was used instead of KBP-088A due to its optimized stability at 37 ˚C suited for infusion (Alzet osmotic pumps, model 2004; AgnTho’s, Lidingö). Male Zucker diabetic fatty (*fa*/*fa*) rats (Charles River Laboratories, Lyon, France) arrived at 6–7 weeks of age and were fed the Purina Laboratory Diet (# 5008, Brogaarden, Denmark) from arrival and throughout the study. The rats developed diabetes before they were allocated to treatment groups on day −2, primarily based on 6 h FBG (average: 19 mM ± 5 SD) and secondarily on body weight (342 g ± 19 SD, *n* = 8 rats/treatment group). The three treatment groups were: vehicle (saline), and KBP-066A delivered by two different approaches: by injection (INJ.) every third day (6 nmol/kg Q3D), or by continuous infusion (INF.) (2 nmol/kg/day). All groups received an injection every 3rd day of either saline solution (vehicle and KBP-066A INF.) or the drug (KBP-066A INJ.) to ensure equal handling. On the day of the study (10–11 weeks old), all rats underwent surgery (vehicle and KBP-066A INF.) or SHAM surgery (KBP-066A INJ.) as described in study (1). The pump was replaced with a new one on day 29 and 57, as described in (1) and the rats receiving INJ underwent SHAM. As the infusion pumps release a fixed amount of drug, all dosing was based on the weight on day −2, 28, and 56 to ensure that KBP-066A INF. vs. INJ. received an equal amount of moles. Food intake and body weight were recorded once daily for one week following surgery/SHAM, then once weekly. FBG (6 h) was measured once weekly. HbA1c was measured at study start and end (11 weeks of treatment), and an OGTT (1 g/kg) was performed after 10.5 weeks of treatment following an 11 h fast.

### 2.8. Oral Glucose Tolerance Test

OGTTs were performed in overnight-fasted rats (16 h in Sprague–Dawley studies and 11 h in ZDF studies). A glucose bolus (1 g/kg for ZDF rats and 2 g/kg for Sprague–Dawley rats) (Sigma-Aldrich, Copenhagen, Denmark) was administered via p.o. gavage at time 0. EDTA blood samples were collected from the tail vein before the glucose challenge (0 min) and then 15, 30, 60, and 120 min post glucose challenge, before being used for insulin measurement. Blood glucose was monitored at time points 0, 15, 30, 60, 120, and 180 min post glucose challenge. S.c. dosing was performed 24 h before all OGTTs. Rats equipped with an infusion pump received continuous dosing throughout the OGTT. 

### 2.9. Intravenous Glucose Tolerance Test (Only Study (4))

IV-GTTs were performed in overnight (16 h)-fasted rats. Glucose (0.6 g/kg) (Sigma-Aldrich, Copenhagen, Denmark) was administered intravenously into the tail vein at time point 0. In the IV-GTT performed after 21 days of treatment, dosing was administered −35 min before the glucose challenge. EDTA blood samples were collected from the tail vein at time −35 min before the glucose challenge, at time point 0 after the glucose challenge, and 15, 30, 60, and 120 min post glucose challenge before being used for insulin measurement. Blood glucose was monitored at time points −35, 0, 15, 30, 60, 120, and 180 min. In the IV-GTT performed after 37 days of treatment, dosing was administered 24 h before the glucose challenge. EDTA blood samples were collected as just described except for the −35 min blood sample. Note, that all rats received 4.5 nmol/kg of KBP-088 or KBP-088A prior to the IV-GTT to prevent dose differences from influencing the test results. 

### 2.10. Biochemical Analysis 

Blood samples were collected in EDTA tubes and centrifuged at 5000 rpm for 10 min at 4 °C and plasma was kept at −20 °C until further analysis. Blood glucose was monitored by the Accu-Check^®^ Avia monitoring system (Roche Diagnostics, Rotkreuz, Switzerland). Plasma insulin was analysed according to the manufacturer’s instructions (Mercodia Rat Insulin ELISA, Mercodia AB, Uppsala, Sweden). HbA1c was measured from the tail vein and analysed using the DCA Vantage Analyzer (Siemens, Erlangen, Germany).

### 2.11. Statistical Analysis

All analyses were performed using GraphPad Prism 8 (GraphPad Software, San Diego, CA, USA). Group differences were assessed using one-way ANOVA followed by Tukey’s post-hoc test for multiple comparison of parametric data. For non-parametric data, Kruskal–Wallis test with Dunn’s post hoc test was applied. Normality of data distribution was determined by the D’Agostino & Pearson (omnibus K2) normality test or the Shapiro–Wilk test if *n* < 8. All data are represented as mean with standard error of the mean (SEM). except bodyweight and FBG data at group allocation which is described with standard deviation (SD). A value of *p* < 0.05 was considered statistically significant. Of note, there is no differentiation between levels of significance. 

## 3. Results

### 3.1. Infusion and Injection of KBP Was Equally Efficient on Weight Loss and Glucose Tolerance in HFD-Fed 

#### Sprague–Dawley Rats (Study (1))

**Figure 2:**
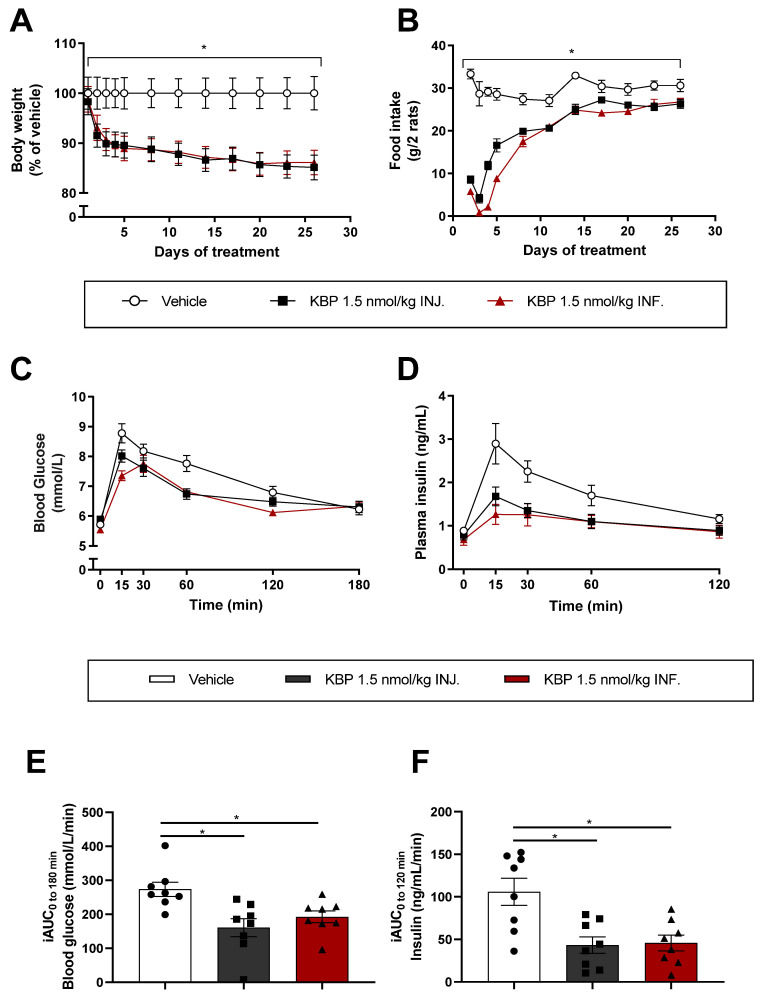
Infusion and injection of KBP in Sprague–Dawley rats. The rats were treated with KBP 1.5 nmol/kg/day delivered by s.c. injection (INJ) or continuous infusion (INF). (**A**) Body weight and (**B**) food intake were monitored once daily for 5 days, then every 3rd day. At study end (day 26), the rats were overnight (16 h) fasted and an OGTT was conducted. The rats were dosed 24 h prior to the glucose challenge. (**C**) Blood glucose and (**D**) plasma insulin were determined during the OGTT, as were the (**E**) iAUC of blood glucose and (**F**) iAUC of plasma insulin during the OGTT. Statistical analysis of tAUC of A) and B) was performed by one-way ANOVA with Tukey’s multiple comparison test. Statistical analysis in (**E**) and (**F**) was performed with one-way ANOVA with Tukey’s multiple comparison test. * *p* < 0.05 treatment(s) compared to vehicle. *n* = 8/group. Data are shown as mean ± SEM. Abbreviations: HFD—high-fat diet. INF.—(drug delivery by) infusion. INJ.—(drug delivery by) injection. tAUC—total area under the curve.

To investigate if the bodyweight-reducing effect of KBP treatment was sensitive to differences in dosing profile, we delivered KBP by injection (INJ.) once daily or by continuous infusion (INF). KBP delivered by INJ. and INF. reduced body weight significantly by 15% ± 4 and 14% ± 4 (SEM) compared to the vehicle (Figure 2A), with no difference between the two treatment groups. Both INJ. and INF. significantly reduced food intake, especially in the initial phase of the study (Figure 2B). The OGTT performed at the study’s end showed that both INJ. and INF. improved glucose tolerance, reducing plasma insulin levels to the same extent (Figure 2C–F). Thus, KBP was equally efficient in terms of body weight and glucose tolerance regardless of the dosing profile. 

### 3.2. Injection of KBP Was Superior to Infusion on Glucose Control in Diabetic ZDF Rats (Study (2))

**Figure 3:**
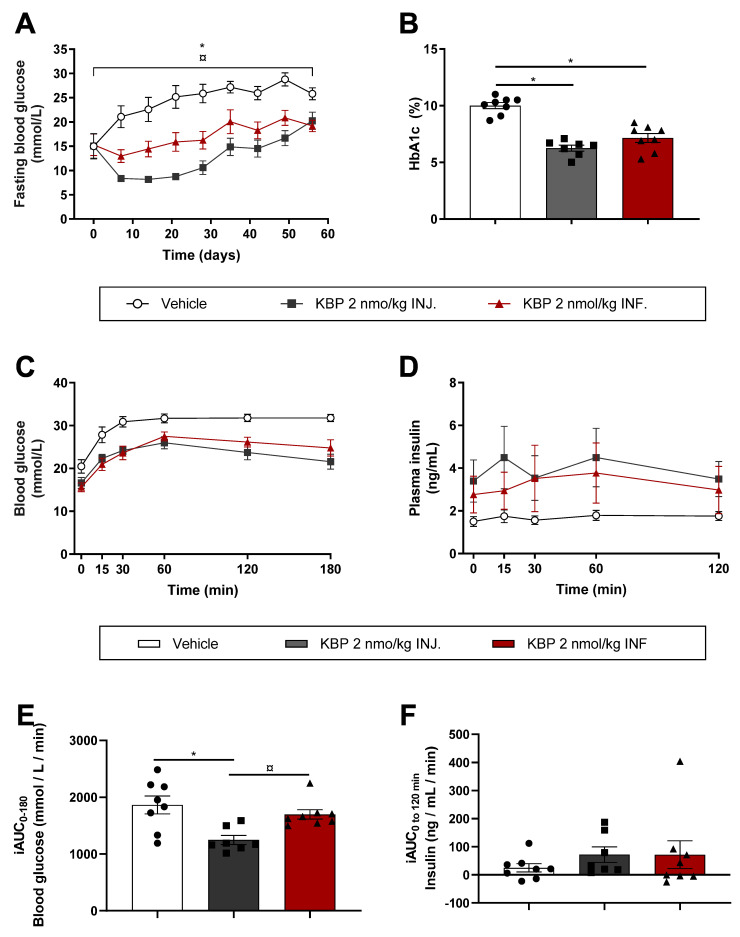
Infusion and injection of KBP in diabetic ZDF rats. The rats were treated with KBP 2nmol/kg delivered by s.c. injection (INJ) or continuous infusion (INF). (**A**) Fasting (6 h) blood glucose was measured weekly, (**B**) and HbA1c at the study end. After 7.5 weeks, the rats were overnight fasted (11 h) before an OGTT. The rats were dosed 24 h prior to the glucose challenge. (**C**) Blood glucose and (**D**) plasma insulin during the OGTT were assessed, (**E**) as were iAUC of blood glucose and (**F**) iAUC of plasma insulin during the OGTT. Statistical analysis of tAUC of (**A**) was performed by one-way ANOVA with Tukey’s multiple comparison test. (**B**) was analysed with one-way ANOVA and Tukey’s multiple comparison test. E) and F) were analysed with Kruskal–Wallis and Dunn’s multiple comparison test. * *p* < 0.05 treatment(s) compared to vehicle. ¤ *p* < 0.05 KBP 2 nmol/kg INJ. compared to INF. *n* = 8/group, except KBP INJ. in which *n* = 7 Data are shown as mean ± SEM. Abbreviations: HbA1c—Hemoglobin A1c. iAUC—ncremental area under the curve. INF—(drug delivery by) infusion. INJ—(drug delivery by) injection. OGTT—oral glucose tolerance test. tAUC—total area under the curve. ZDF—Zucker diabetic fatty.

We next examined the influence of the KBP dosing profile on glucose control in diabetic rats by delivering KBP via INJ. or INF. Both INJ. and INF. significantly lowered FBG by tAUC during the study period as well as HbA1c measured at study end (Figure 3A,B). Interestingly, delivery by INJ. was superior to INF. by tAUC in terms of lowering FBG, although the difference evened out at the study end (Figure 3A). Following 7.5 weeks of treatment, when the treatment effect on FBG was similar in the two groups, the rats underwent an OGTT. KBP INF. and INJ. significantly improved glucose tolerance by lowering blood glucose by tAUC (Figure 3C and Supplementary Figure S6A) though only KBP INJ. lowered blood glucose significantly by iAUC (Figure 3E). This was possibly mediated by a significantly higher insulin secretion in the KBP INJ. group when assessed by tAUC (Figure 3D and Supplementary Figure S3B) but not iAUC (Figure 3F). As expected, KBP treatment did not change the body weight significantly [13,16] (Supplementary Table S1). 

### 3.3. Both KBP and KBP-A Improve Body Weight and Glucose Tolerance in HFD-Fed Sprague–Dawley Rats (Study (3) and (4)) 

**Figure 4:**
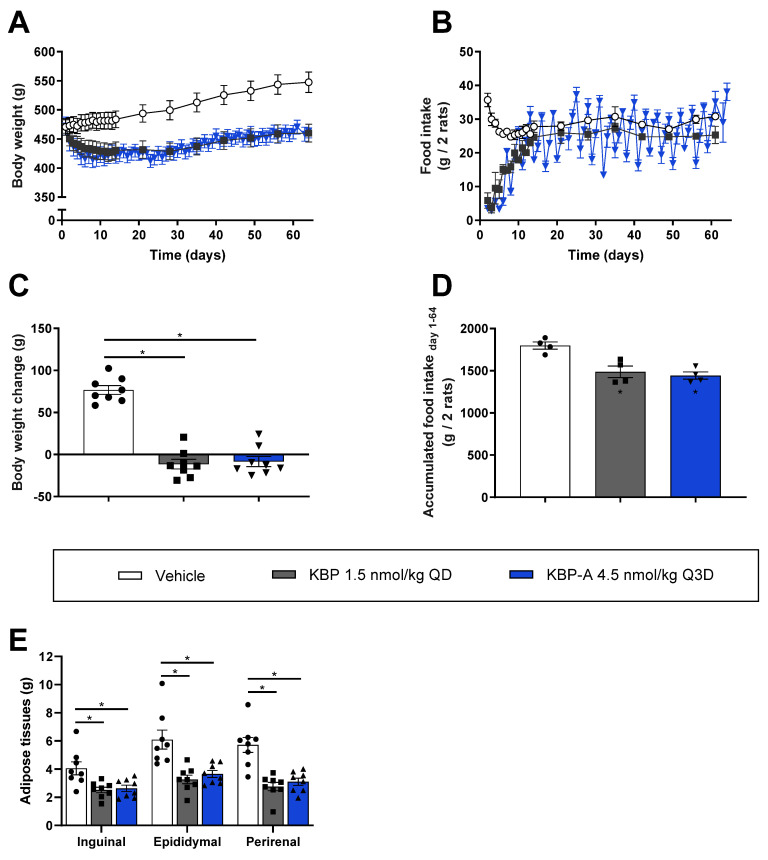
Comparison of KBP dosed daily (1.5 nmol/kg QD) and KBP-A dosed every 3rd day (4.5 nmol/kg Q3D) in HFD Sprague–Dawley rats. (**A**) Body weight and (**B**) food intake during the study. (**C**) Body weight at study end and (**D**) accumulated food intake. (**E**) Weight of adipose tissues (inguinal, epididymal, and perirenal) at study end). *n* = 8/group. Statistical analyses were performed with one-way ANOVA and Tukey’s multiple comparisons test. * *p* < 0.05 treatment(s) compared to vehicle. Data are shown as mean ± SEM. Abbreviations: AT—adipose tissue. HFD—high-fat diet. QD—once daily. Q3D—once every 3rd day.

**Figure 5:**
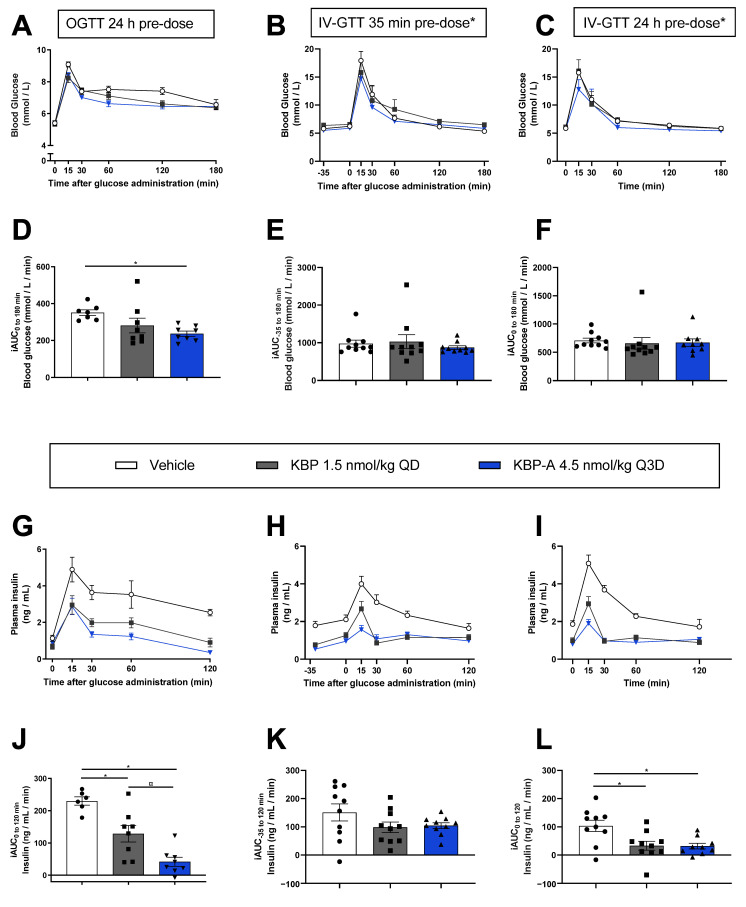
Assessment of glucose tolerance in overnight fasted (16 h) HFD Sprague–Dawley rats treated with saline (vehicle), KBP dosed once daily (1.5 nmol/kg QD), KBP-A dosed every 3rd day (4.5 nmol/kg Q3D). Three tests were performed: an OGTT (**A**,**D**,**G**,**J**) and two IV-GTT performed following 8 weeks, 3 or 5 weeks of treatment, where the last dose was s.c. injected 24 h, 35 min (**B**,**E**,**H**,**K**), or 24 h (**C**,**F**,**I**,L) prior to the glucose challenge. The OGTT was performed in one study, while the two IV-GTT were performed in other rats in a separate study. Graphs (**A**,**B**,**C**) show glucose levels during the tests and the calculated incremental area under the curves (iAUC) (**D**,**E**,**F**), while graphs (**G**,**H**,**I**) show plasma insulin levels during the tests and the calculated iAUCs (**J**,**K**,**L**). Pre-dose *: Rats receiving KBP were dosed with 4.5 nmol/kg, not 1.5 nmol/kg, prior to the IV-GTT to ensure that the results were not affected by acute dose differences. Pre-dosing 35 min favored the rapid plasma peak of KBP and 24 h pre-dosing favored the slow plasma peak of KBP-A. *n* = 8/group in the OGTT except for the vehicle where *n* = 7. *n* = 8/group in the 35 min pre-dose IV-GTT. *n* = 8/group in the 24 h pre-dose IV-GTT, except in the KBP-A group where *n* = 7. Statistical analysis of (**D**,**J**,**K**,**L**) were performed with one-way ANOVA and Tukey’s multiple comparisons test, and (**E**,**F**) were analysed via the Kruskal–Wallis and Dunn’s multiple comparison tests. * *p* < 0.05 treatment(s) compared to vehicle and ¤ *p* < 0.05 compared to KBP. Data are shown as mean ± SEM. Abbreviations: HFD—high-fat diet. IV-GTT—intravenous glucose tolerance test. OGTT—oral glucose tolerance test. Q3D—once every 3rd day. QD—once daily.

The impact of the different pharmacodynamic (PD) profiles of KBP and KBP-A on in vivo efficacy was explored in HFD Sprague–Dawley rats. The long-acting KBP-A was dosed every 3rd day (Q3D) which corresponds to once-weekly dosing in humans [24,25]. KBP dosed QD and KBP-A dosed Q3D significantly reduced bodyweight (16% ± 4 and 15% ± 4 SEM) and overall adiposity compared to vehicle but without differentiating from one another (Figure 4A,C–E). This was expected and in line with a previous 4-week study using KBP and KBP-A [16]. Food intake was markedly suppressed by KBP and KBP-A in the initial treatment phase but normalized on day 10–14 (Figure 4B). However, the food intake of KBP-A fluctuated throughout the study period thereby reflecting the Q3D dose interval and exposure profile. During the OGTT performed at study end, only KBP-A lowered blood glucose significantly compared to vehicle (Figure 5A,D). The insulin lowering effect was significant for both KBP and KBP-A, albeit more pronounced for KBP-A which also lowered insulin significantly compared to KBP (Figure 5G,J). Thus, KBP-A was superior to KBP in terms of oral glucose tolerance. Next, for optimal comparison of KBP and KBP-A on glucose tolerance, we conducted a separate study (study (4)) with two IV-GTTs to exclude the drug-induced and dose-dependent reduction of gastric emptying. Importantly, the last dose before the IV-GTTs was 4.5 nmol/kg regardless of the “normal” dose concentration. Bodyweight and food intake from the study are shown in Supplementary Figure S7. but reproduced that of study (3) (Figure 4A,B). In both IV-GTTs, regardless of whether KBP and KBP-A had been dosed 35 min or 24 h before the glucose challenge, blood glucose did not differ from vehicle (Figure 5B,C,E,F, and Supplementary Figure S8A,B). In contrast, KBP and KBP-A significantly lowered insulin by tAUC compared to vehicle in both IV-GTTs (Figure 5H,I,K,L, and Supplementary Figure S8C,D). Thus, when bypassing the gastric emptying effect and using equimolar doses, KBP and KBP-A improved glucose tolerance equally well. 

### 3.4. KBP-A Improves Glucose Control in Diabetic ZDF Rats Independent of Dosing Profile (Study (5))

**Figure 6:**
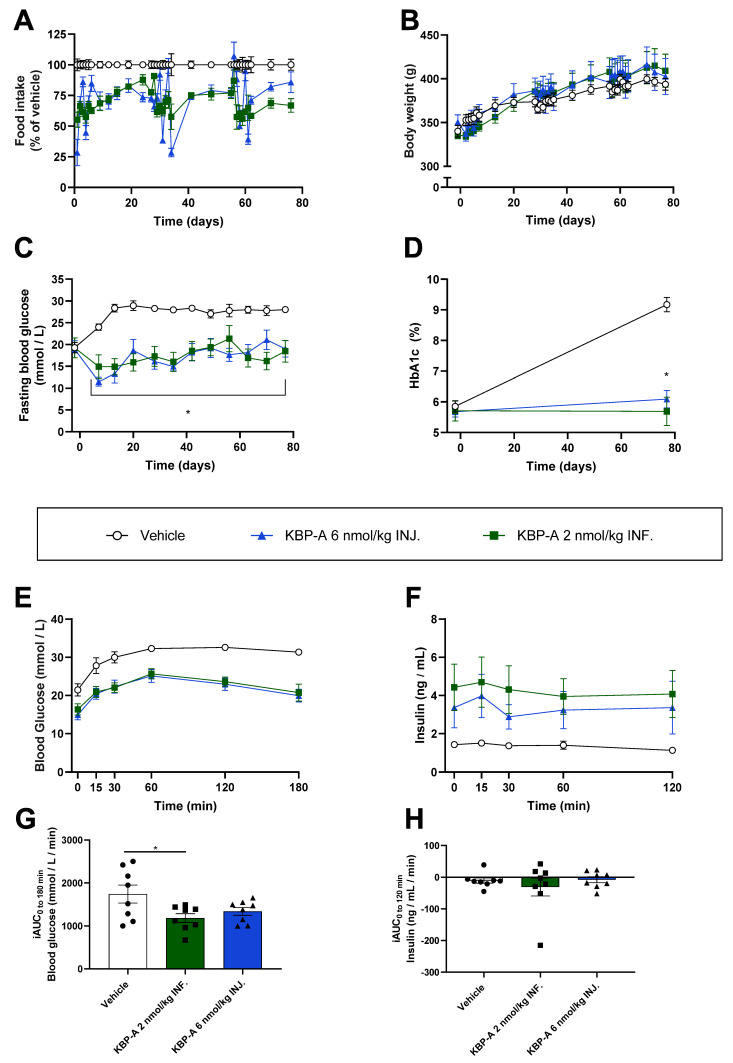
Intervention study in ZDF rats treated with saline, or KBP-A delivered by continuous infusion (INF., 2 nmol/kg QD) or by injection every 3rd day (INJ., 6 nmol/kg Q3D). The rats underwent surgery or SHAM when the infusion pump was inserted. Food intake (**A**) and body weight (**B**) were measured daily following surgery/SHAM, then once weekly. The pump was replaced on day 29 and day 57. (**C**) FBG (6 h) was measured once weekly and (**D**) HbA1c was measured at baseline and study end. After 10.5 weeks, the rats were overnight fasted (11 h) before an OGTT. KBP-A INJ. rats were dosed 24 h prior to the glucose challenge, while KBP-A INF. rats received continuous dosing during the OGTT due to the nature of the infusion pump. (**E**) Blood glucose and (**F**) plasma insulin were measured during the OGTT, as were (**G**) iAUC of blood glucose and (**H**) iAUC of plasma insulin during the OGTT. *n* = 8/group. Statistical analysis of tAUC of (**C**) was evaluated by Kruskal–Wallis test with Dunn’s multiple comparisons test. (**D**) was evaluated by two-way ANOVA with Tukey’s multiple comparison. ((**G**) was evaluated by one-way ANOVA with Tukey’s multiple comparisons test. * *p* < 0.05 treatment(s) compared to vehicle. Data are shown as mean ± SEM. Abbreviations: FBG—fasting blood glucose. HbA1c—Hemoglobin A1c. iAUC—incremental area under the curve. INF—(drug delivery by) infusion. INJ.—(drug delivery by) injection. OGTT—oral glucose tolerance test. QD—once daily. Q3D—once every 3rd day. tAUC—total area under the curve. ZDF—Zucker diabetic fatty.

Our previous studies (study (2) and [16]) had shown that a fluctuating PD profile benefitted glucose control in diabetic ZDF rats. We therefore hypothesized that the superiority of KBP-A compared to KBP on glucose control was due to the extended and fluctuating PD profile. To investigate this, we eliminated the fluctuations in PK and PD by delivering KBP-A by INF. (2 nmol/kg QD) and compared it to INJ. (6 nmol/kg Q3D) in diabetic ZDF rats. KBP-A INJ. resulted in a fluctuating food intake after each injection, reflecting the exposure profile (Figure 6A). Of note, food intake was only measured daily following pump insertion/SHAM otherwise weekly. As expected [13,16], KBP-A treatment did not change the body weight significantly (Figure 6B). Both INJ. and INF. suppressed FBG significantly and to the same extent as was also reflected in HbA1c (Figure 6C,D). Similarly, both INJ. and INF. significantly lowered blood glucose and preserved insulin secretion (by tAUC) during the OGTT compared to the vehicle. However, only INF. lowered blood glucose significantly by iAUC (Figure 6E–H, Supplementary Figure S9). 

## 4. Discussion

The effect of the GPCR class B ligand, PTH, heavily depends on the exposure profile. Since KBP and KBP-A activate receptors belonging to the same family, we investigated whether this also applied to KBP and KBP-A. We investigated weight reduction and glucose tolerance in HFD Sprague–Dawley rats, and FBG and glucose tolerance in diabetic ZDF rats. Here we conclude that different dosing profiles of KBP (INJ. and INF.) and KBP-A (INJ.) produce an equipotent weight loss and food suppression in HFD rats. Though KBP-A was superior to KBP in terms of oral glucose tolerance, this was due to a dose-dependent and drug-induced reduction of the gastric emptying rate, consistent with previous data [16,26], and could be overcome by dosing equimolarly and delivering the glucose load IV. In contrast, in diabetic ZDF rats, the dosing profile of KBP, when delivered by INJ., seemed to benefit glucose control to a greater extent than when performed by INF. However, the superiority of the INJ. dosing profile declined as the genetic driver of the disease progression made FBG more difficult to control. We then compared our data to the findings by [16], who demonstrated that KBP-A was superior to KBP in ZDF rats with regards to lowering FBG, HbA1c, and preserving insulin secretion. Based on this, we hypothesized that a fluctuating exposure profile was beneficial for the efficacy of KBP-A in T2D treatment [16]. However, INJ. vs. INF. of KBP-A performed equally well on FBG, HbA1c, and oral glucose tolerance. This would indicate that PD matters for KBP but not KBP-A, possibly due to differences between the two peptides (presence or absence of acylation) causing a pharmacological difference. Treatment efficacy obtained by injection versus continuous infusion has also been investigated for the glucagon-like peptide-1 (GLP-1) receptor agonist, exenatide, of which the injectable version is approved as treatment for patients with inadequately controlled type 2 diabetes. Continuously infused exenatide aimed to improve patient convenience and adherence to treatment. The clinical studies show that exenatide produced the same reduction in fasting plasma glucose and HbA1c when delivered by continuous infusion or twice-daily injections [27]. We speculate that the similar treatment effect of KBP-A INJ. and INF. could be related to the dose concentration, which was aimed to be the maximum efficacious dose [15,16]. Small differences between INF. and INJ. may have gone undetected due to optimal dose concentration. Using half-optimal dosing instead may have revealed a difference in INJ. and INF. efficacy. However, when KBP-A is dosed optimally, both INF. and INJ. are equally relevant from a clinical perspective. 

Although an acylated amylin analog is now in clinical trials, there are no head-to-head comparison studies of daily versus weekly dosed amylin analogs or DACRAs [28,29]. Other drug classes are therefore relevant as references e.g., glucagon-like peptide 1 receptor (GLP-1R) agonists since both daily or twice daily versus weekly dosed peptides have been tested head-to-head in terms of the weight-reducing and glucoregulatory effect. In a direct comparison of the almost identical liraglutide (1.2 mg daily) and semaglutide (1.0 mg weekly), semaglutide was superior in terms of HbA1c and weight reduction, although the liraglutide dose used was in the lower end of the FDA-approved doses [30]. Similarly, weekly-dosed exendin-4 is superior to exendin-4 given twice daily in terms of HbA1c reduction but induce similar weight loss [31,32,33,34]. These examples underline that weekly dosed peptides are likely to exceed their daily or twice daily dosed counterparts, although the examples do not provide insight into whether superiority is due to the exposure profile. Furthermore, the comparison of exendin-4, dosed weekly versus twice daily, supports our observation of a benefit for glucose control but not weight reduction when dosing less frequently [31,32,33,34]. 

In summary, we found that the different dosing profiles of KBP and KBP-A were equally potent in terms of weight reduction and post-prandial glucose control in HFD rats. In contrast, in diabetic ZDF rats, the injectable dosing profile of KBP appeared to control FBG and possibly insulin secretion better than infusion, while injection and infusion with KBP-A resulted in similar glucose control. In conclusion, different dosing profiles of KBP and KBP-A are equipotent in terms of weight loss, while there appear to be small differences in the potency order (low-to-high) when treating type 2 diabetes: KBP infusion < KBP injection < KBP-A injection = KBP-A infusion.

## Figures and Tables

**Table 1:** Amino acid sequence of KBP-042, KBP-088, KBP-088A, and KBP-066A. The peptides consist of an NH_2_-terminal acetyl group (Ac), and a COOH-terminal amide group. KBP-088A and -066A have an additional acylation conjugated to the lysine (shown in bold). The chemical structure of the acylation is shown at the bottom of the table.

KBP-042: *Ac-*CSNLSTCVLGKLSQELHKLQTYPRTDVGANAP*-NH2* KBP-088: *Ac-*CSNLSTCMLGRLSQELHRLQTFPKTDVGANAP*-NH2* KBP-088A: *Ac-*CSNLSTCMLGKLSQELHRLQTFPKTDVGANAP*-NH2* KBP-066A: *Ac-*CSNLSTCXLGKLSQDLHRLQTYPKTDVGANAP*-NH2*
***Ac***: Acetylation of the N-terminus ***X***: AiB (2-Aminoisobutyric acid) ***K***: -PEG-PEG-γGlu-C18 diacid acylation complex conjugated to the lysine side chain ***NH2***: Amidation of the C-terminal COOH group
PEG-PEG-yGlu-C18 diacid 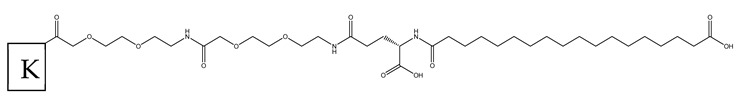

## Data Availability

The datasets used and/or analysed during the current studies are available from the corresponding author upon reasonable request.

## Supplementary Materials

The following supporting information can be downloaded at: https://www.mdpi.com/article/10.3390/biomedicines10102365/s1, Figure S1: Schematic diagrams of the in vivo study designs; Figure S2: Stability of KBP-042 (20 µM) in mannitol (5%, pH 4) matrix at day 1, 7, 14, and 28 at 37 ˚C. Stability; Figure S3: Stability of KBP-088 (90 µM) in saline matrix at day 0 and 28 at 37 ˚C; Figure S4: Biological activity of KBP-066A; Figure S5: Stability of KBP-066A (1000 µM) in saline matrix at day 0 and 28 at 37 ˚C; Figure S6: Diabetic ZDF rats were treated with KBP 2 nmol/kg delivered by s.c. injection (INJ) or continuous infusion (INF); Figure S7: Body weight and food intake in HFD Sprague-Dawley rats undergoing IV-GTTs; Figure S8: tAUC of IV-GTTs in HFD Sprague-Dawley rats; Figure S9: tAUC of an OGTT in diabetic ZDF rats following 10.5 weeks treatment with vehicle (saline); Table S1: Body weight of diabetic ZDF rats during study.

## Abbreviations

AMY-R:	amylin receptor
CTR:	calcitonin receptor
DACRA:	Dual amylin and calcitonin receptor agonist
FBG:	fasting blood glucose
GPCR:	G protein-coupled receptor
KBP:	KeyBiosciencePeptide
HFD:	high-fat diet
INF.:	(drug delivery by) infusion
INJ.:	(drug delivery by) injection
IV:	intravenous
IV-GTT:	intravenous glucose tolerance test
OGTT:	oral glucose tolerance test
QD:	once daily
Q2D:	once every 2nd day/once every other day
Q3D:	once every 3rd day
RAMP:	receptor activity-modifying protein
s.c.:	subcutaneous
ZDF:	Zucker Diabetic Fatty (rat)
