# Supplementary material for: The Impact of Exposure Profile on the Efficacy of Dual Amylin and Calcitonin Receptor Agonist Therapy
Source: Biomedicines. 2022 Sep 22;10(10):2365. doi: 10.3390/biomedicines10102365 (PMC9599033; doi:10.3390/biomedicines10102365)
Supplement: Supplementary file 1 [file biomedicines-10-02365-s001.zip › biomedicines-1895024-supplementary.pdf]

Supplemental data

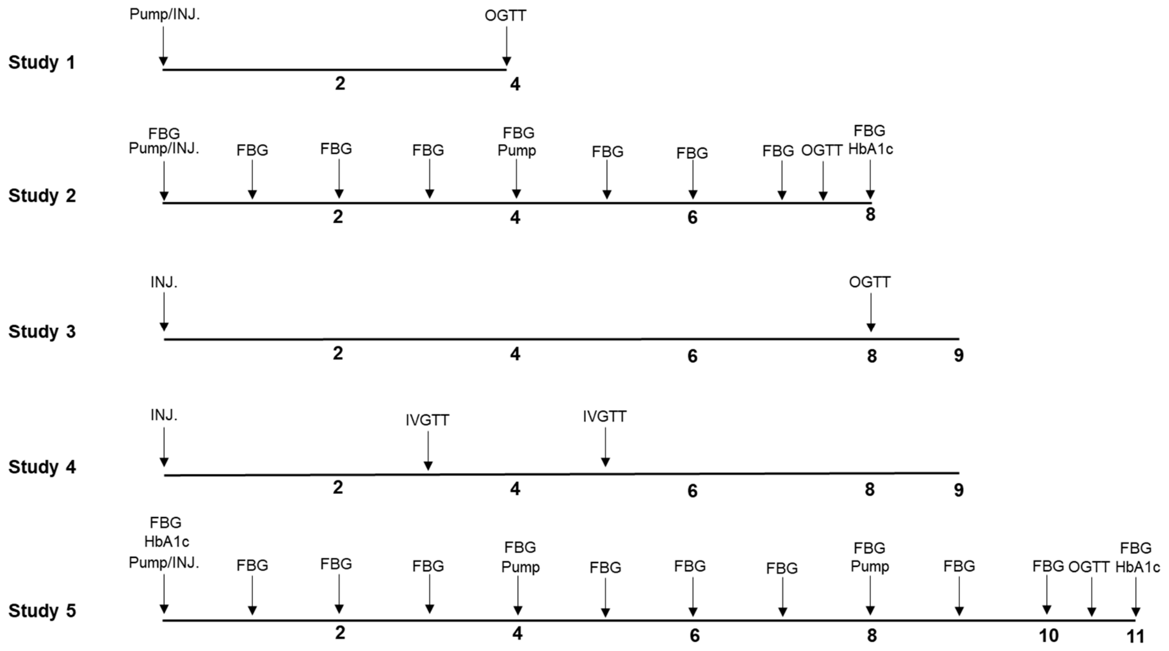

Suppl. Figure S1. Schematic diagrams of the *in vivo* study designs. **Study 1**) Infusion versus injection of KBP-042 in HFD-fed Sprague-Dawley rats. **Study 2**) Infusion vs. injection of KBP-088 in diabetic ZDF rats. **Study 3**) Comparison of KBP-088 and KBP-088A in HFD-fed Sprague-Dawley rats. **Study 4**) Intravenous glucose tolerance test in HFD-fed Sprague-Dawley rats. **Study 5**) Infusion vs. injection of KBP-066A in diabetic ZDF rats. FBG: fasting blood glucose, HbA1c: glycated haemoglobin A1c, INJ: injection, IVGTT: intravenous glucose tolerance test, OGTT: oral glucose tolerance test, Pump: insertion of new infusion pump.

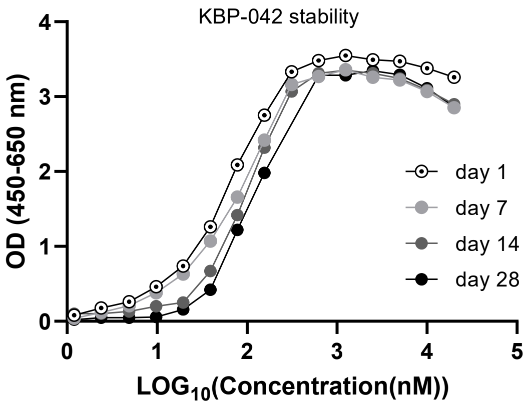

Suppl. Figure S2. Stability of KBP-042 (20 µM) in mannitol (5%, pH 4) matrix at day 1, 7, 14, and 28 at 37˚C. Stability was assessed by an in-house developed ELISA assay. Data is shown as the mean of duplicates. A max CV of 10% was accepted.

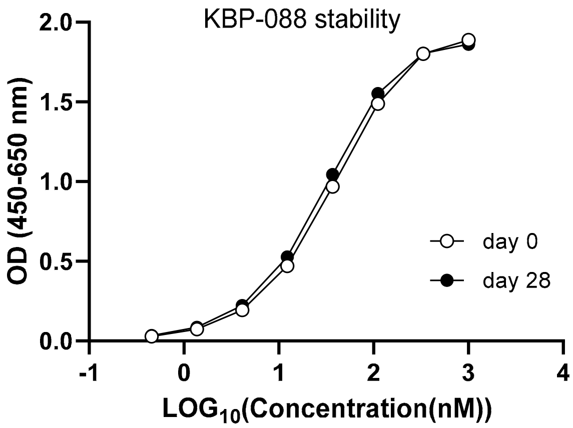

Suppl. Figure S3. Stability of KBP-088 (90 µM) in saline matrix at day 0 and 28 at 37˚C. Stability was assessed by an in-house developed ELISA assay. The chosen 90 µM concentration was the minimum concentration used in the osmotic infusion pumps in ZDF rats. Data is shown as the mean of duplicates. A max CV of 10% was accepted.

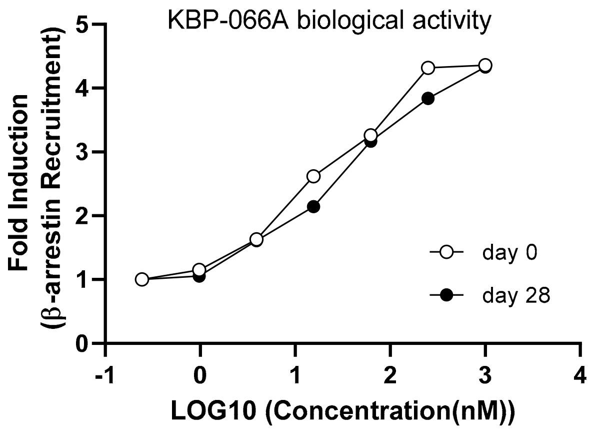

Suppl. Figure S4. Biological activity of KBP-066A. KBP-066A (100 µM) was stored at 37˚C and tested at day 0 and 28. The biological activity was tested using a β-arrestin recruitment assay in the CTRa overexpressing cell line U2OS CALCR. Data is shown as the mean of four technical replicates. A max CV of 10% was accepted.

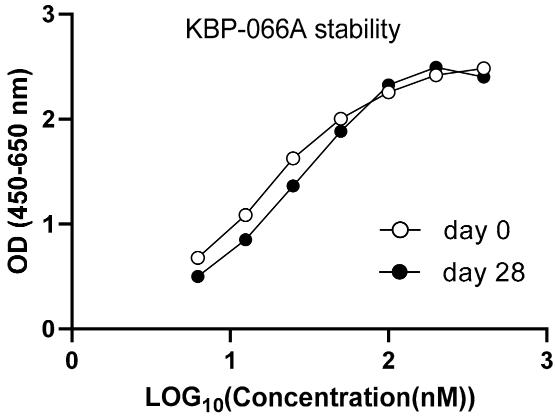

Suppl. Figure S5. Stability of KBP-066A (1000 µM) in saline matrix at day 0 and 28 at 37˚C. Stability was assessed by an in-house developed ELISA assay. Data is shown as the mean of duplicates. A max CV of 10% was accepted.

Suppl. Table S1. Body weight of diabetic ZDF rats during study 2). Abbreviations: QD – once-daily. ZDF – Zucker diabetic fatty

	ZDF (*fa/fa*)		
Treatment<br>Dose<br>Saline/drug delivery	Vehicle<br>-<br>s.c. injection QD (saline)	KBP<br>2 nmol/kg<br>s.c. injection QD (drug)	KBP<br>2 nmol/kg<br>s.c. injection QD (saline) + continuous drug infusion
Day	Weight (g) ± SEM	Weight (g) ± SEM	Weight (g) ± SEM
-1	335 ± 7	338 ± 7	349 ± 5
7	344 ± 7	333 ± 8	340 ± 4
14	357 ± 9	353 ± 10	351 ± 5
21	370 ± 10	370 ± 11	366 ± 7
28	367 ± 13	377 ± 12	375 ± 8
35	371 ± 12	391 ± 12	380 ± 11
42	375 ± 12	396 ± 13	385 ± 11
49	382 ± 12	402 ± 15	398 ± 12
52	379 ± 12	405 ± 15	400 ± 12

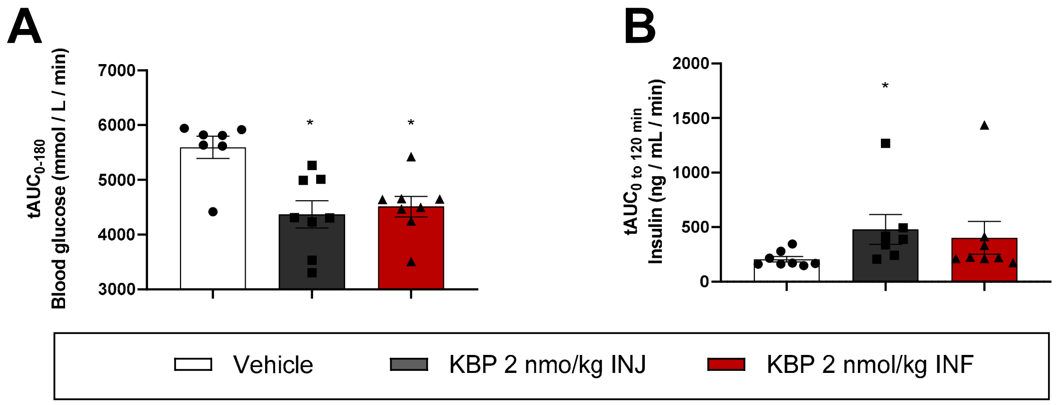

Suppl. Figure S6. Diabetic ZDF rats were treated with KBP 2 nmol/kg delivered by s.c. injection (INJ) or continuous infusion (INF). An OGTT was performed following 7.5 weeks of treatment. The rats were overnight fasted (11 h) and an oral glucose bolus (1 g/kg) was delivered. Blood glucose was measured at timepoint 0 (before glucose), 15, 30, 60, 120 and 180 min post-glucose challenge, and blood samples collected at time point 0, 15, 30, 60 and 120 min for insulin measurement. n = 8/group, except in the KBP-088 INJ group in which n = 7. Statistical analyses of A) and B) was performed with Kruskal-Wallis test and Dunn's multiple comparison test. *P < treatment(s) 0.05compared to vehicle. Data are shown as mean ± SEM. Abbreviations: tAUC – total area under the curve. INF – infusion (of KBP-088). INJ – injection (of KBP-088). OGTT – oral glucose tolerance test. ZDF – Zucker diabetic fatty.

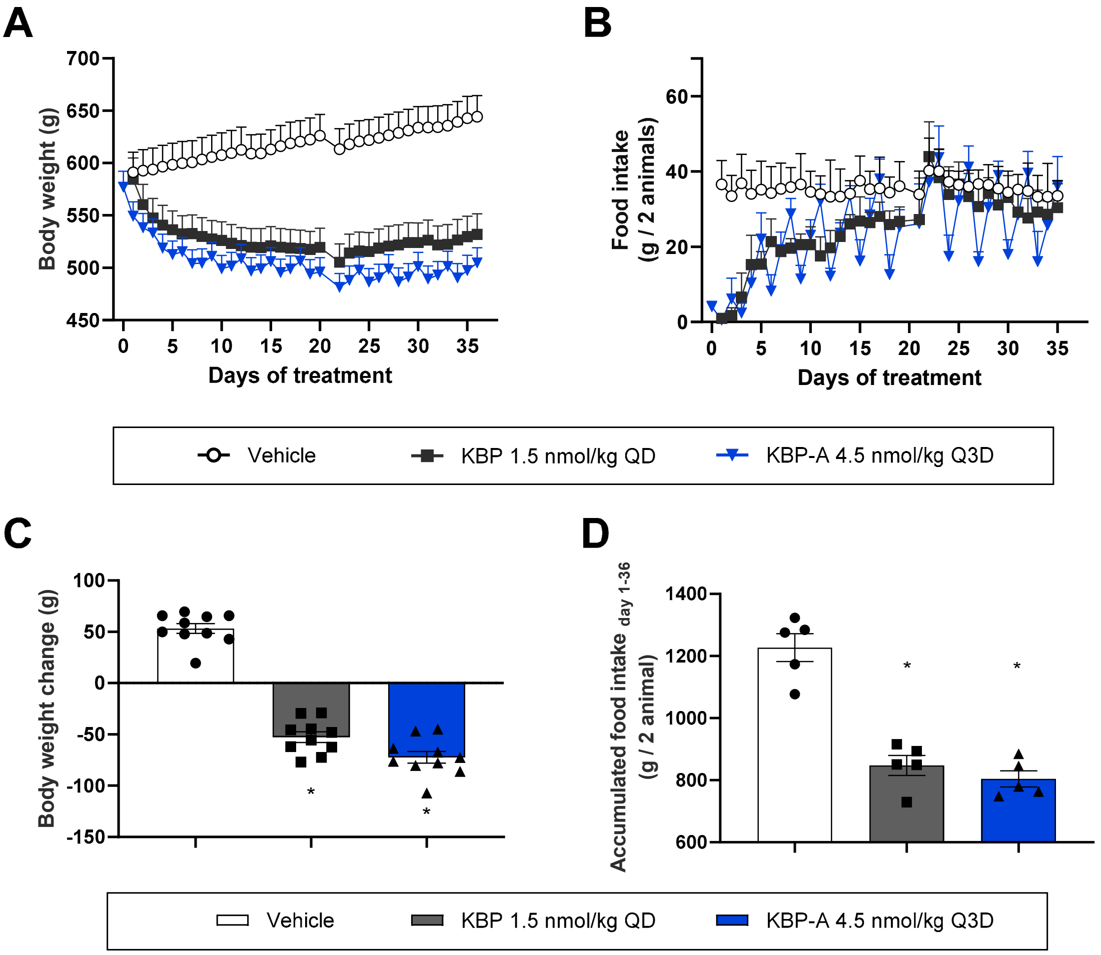

Suppl. Figure S7. Body weight and food intake in HFD Sprague-Dawley rats undergoing IV-GTTs. The rats were treated for 37 days with saline (vehicle), daily KBP (1.5 nmol/kg QD) or KBP-A dosed every 3rd day (4.5 nmol/kg Q3D). A) Body weight, B) and food intake were measured daily. C) Body weight change and D) accumulated food intake were calculated. n = 8/group. Statistical analysis was evaluated by one-way ANOVA with Tukey's multiple comparison test. *P < 0.05 compared to vehicle. Data are shown as mean with SEM. Abbreviations: HFD – high fat diet. IV-GTT – intra-venous glucose tolerance test. QD – once daily. Q3D – once every 3rd day.

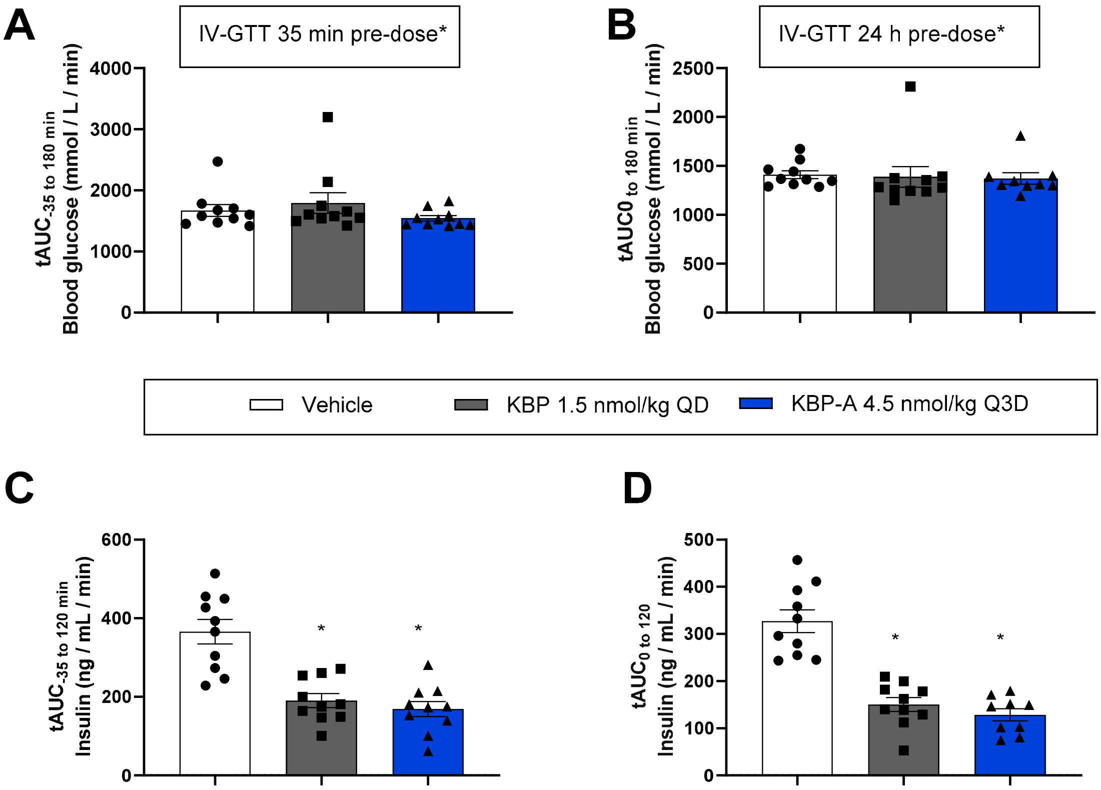

Suppl. Figure S8. tAUC of IV-GTTs in HFD Sprague-Dawley rats. The rats were treated for 37 days with saline (vehicle), daily KBP (1.5 nmol/kg QD) KBP-A dosed every 3rd day (4.5 nmol/kg Q3D). The first IV-GTT (A and C) was performed following 21 days of treatment with dosing administered 35 min before the glucose challenge. The second IV-GTT (B and D) was performed following 37 days of treatment with dosing administered 24 h before the glucose challenge. **Pre-dose*:** KBP-treated rats received 4.5 nmol/kg, not 1.5 nmol/kg, prior to the IV-GTT to ensure that the results were not affected by acute dose-differences. The tAUCs of blood glucose are shown in A) and B) while the tAUCs of insulin are shown in C) and D). n = 8/group. Statistical analysis was evaluated by one-way ANOVA with Tukey's multiple analysis. *$P < 0.05$ compared to vehicle. Data are shown as mean with SEM. Abbreviations: HFD – high fat diet. IV-GTT – intra-venous glucose tolerance test. QD – once daily. Q3D – once every 3rd day. tAUC – total area under the curve.

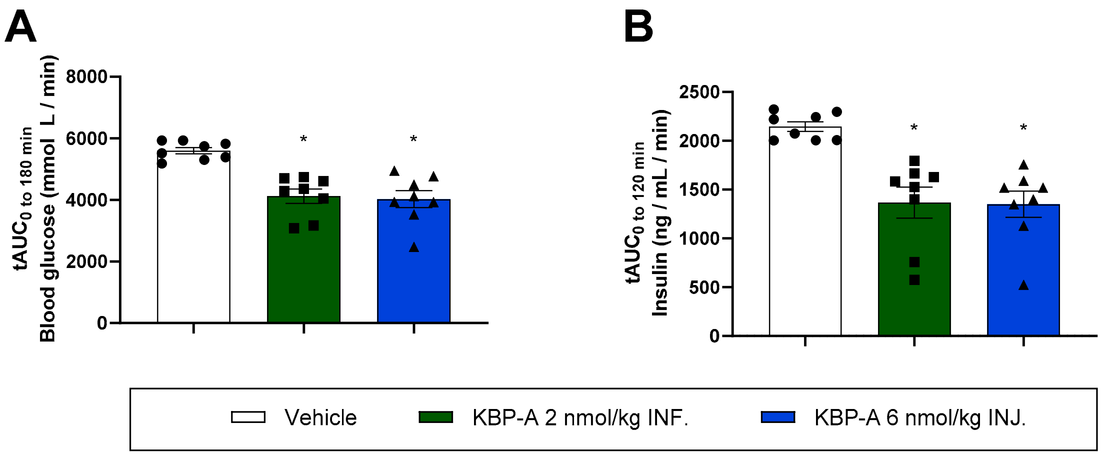

Suppl. Figure S9. tAUC of an OGTT in diabetic ZDF rats following 10.5 weeks treatment with vehicle (saline), KBP-066A delivered by injection every 3rd day (6 nmol/kg Q3D INJ.) or by continuous infusion (2 nmol/kg QD INF.). The last dose in the INJ. group was administered 24 h prior to the test. A) tAUC of blood glucose and B) plasma insulin during the OGTT. n = 8/ group. Statistical analysis was evaluated by one-way ANOVA and Tukey's multiple comparisons test. *$P < 0.05$ compared to vehicle. Data are shown as mean with SEM. Abbreviations: INF. – (drug delivery by) infusion. INJ. – (drug delivery by) injection. OGTT – oral glucose tolerance test. QD – once daily. Q3D – once every 3rd day. tAUC – total area under the curve. ZDF – Zucker Diabetic fatty (rat)

Stability test of KBP-042: ELISA

The stability of KBP-042 was determined by an in-house developed sandwich ELISA. The stability of KBP-042 was tested at 37˚C, following 1, 7, 14, and 28 days of storage from a stock of 20 µM, which was lower than the concentration used in the minipump (~110 µM). Mannitol (5%, pH 4) was used as stock matrix.

The ELISA was performed as described in (Hjuler *et al.*, 2015) with some modifications specified in the following. Briefly, the in-house sandwich ELISAs was developed using two in-house antibodies targeting the N-terminal (HRP-labeled detector antibody) and C-terminal end (Biotinylated coater antibody) of KBP-042. KBP-042 was used as calibrator (20,000-0 nM, 2-fold dilution).

KBP-042 stability samples were diluted in mannitol (5%, pH 4) in a 2-fold dilution series identical to

the calibrator. Test samples and calibrator were measured in duplicate. Non-peptide materials, volumes, and times were as described in (Hjuler *et al.*, 2015).

Stability test of KBP-088: ELISA

The stability of KBP-088 was tested at 37˚C, following 0 and 28 days of storage from a stock of 90 μM which was the lowest concentration used in the minipumps used in study 2. KBP-088 was used as calibrator and saline (0.9%) was used as matrix.

The in-house ELISA assay was performed as described for KBP-042 with some modifications. The same antibodies were used for KBP-088, since KBP-042 and -088 shares N- and C-terminus. The KBP-088 ELISA used 200 ng/mL coater and 100 ng/mL detector, and a 400-0.5 nM calibrator range (3-fold dilution) using buffer as diluent. KBP-088 stability samples were diluted in buffer to 1 μM and then diluted in a 3-fold dilution series (1000-0.46 nM).

Stability test of KBP-066A: ELISA

The stability of KBP-066A was tested at 37˚C, following 0 and 28 days of storage from a stock of 1000 μM.. KBP-066A was used as calibrator and saline (0.9%) was used as matrix.

The in-house ELISA assay was performed as described for KBP-042 and -088 with some modifications. KBP-066A also shares N- and C-terminus with KBP-042. The KBP-066A ELISA used 400 ng/mL coater and 800 ng/mL detector, and a 400-6.25 nM calibrator range (2-fold dilution) using buffer as diluent. The KBP-066A stability samples were diluted in buffer to 400 nM and then diluted in a 2-fold dilution series identical to the calibrator.

Activity and stability test of KBP-066A: Cell-based assay

The stability of KBP-066A was determined by a cell-based assay which measures biological activity, as well as the above-described sandwich ELISA. The stability of KBP-066A was tested at 37˚C

following 0 and 28 days of storage from a stock of 100 μM which was the approximate concentration used in the minipumps used in study 5. Saline (0.9%) was used as matrix. Peptide activity was performed using a β-arrestin recruitment assay in the CTRa overexpressing cell line U2OS CALCR as described in (Andreassen, Hjuler, *et al.*, 2014).
